# Harmonic Scalpel versus Conventional Haemostasis in Neck Dissection: A Prospective Randomized Study

**DOI:** 10.1155/2013/369345

**Published:** 2013-12-22

**Authors:** Emanuele Ferri, Enrico Armato, Giacomo Spinato, Marcello Lunghi, Giancarlo Tirelli, Roberto Spinato

**Affiliations:** ^1^Otorhinolaryngology Department ULSS 17, General Hospital of Monselice, Via G. Marconi 19, Monselice, 35043 Padua, Italy; ^2^Otorhinolaryngology Department ULSS 13, General Hospitals of Dolo and Mirano, Via Mariutto 76, Mirano, 30035 Venice, Italy; ^3^Head and Neck Department, ENT Clinic, Hospital of Cattinara, University of Trieste, Strada di Fiume 447, 34149 Trieste, Italy

## Abstract

*Purpose.* The aim of this prospective randomized trial was to compare operative factors, postoperative outcomes, and surgical complications of neck dissection (ND) when using the harmonic scalpel (HS) versus conventional haemostasis (CH) (classic technique of tying and knots, resorbable ligature, and bipolar diathermy). *Materials and methods. * Sixty-one patients who underwent ND with primary head and neck cancer (HNSCC) resection were enrolled in this study and were randomized into two homogeneous groups: CH (conventional haemostasis with classic technique of tying and knots, resorbable ligature, and bipolar diathermy) and HS (haemostasis with harmonic scalpel). Outcomes of the study included operative time, intraoperative blood loss, drainage volume, postoperative pain, hospital stay, and incidence of intraoperative and postoperative complications. *Results.* The use of the HS reduced significantly the operating time, the intraoperative blood loss, the postoperative pain, and the volume of drainage. No significant difference was observed in mean hospital stay and perioperative, and postoperative complications. *Conclusion.* The HS is a reliable and safe tool for reducing intraoperative blood loss, operative time, volume of drainage and postoperative pain in patients undergoing ND for HNSCC. Multicenter randomized studies need to be done to confirm the advantages of this technique and to evaluate the cost-benefit ratio.

## 1. Introduction

Major otolaryngology procedures are often complicated by challenging anatomy, complex reconstructions, and long operative times. Furthermore, many patients undergoing these treatments have comorbid medical conditions that complicate their care and may cause perioperative complications. Neck dissection (ND) has been recognized as an integral part of the surgical therapy of head and neck cancer since the 19th century [1, 2]. Since then, many technical changes have been proposed; in particular, it has been modified to preserve vital vascular and nervous structures while maintaining its therapeutic efficacy. ND is commonly used in the treatment of cervical lymphatic metastases of malignant disease of the upper aerodigestive tract, thyroid, parotid, and skin of the head and neck. Although the rate of complications following ND accounts from 6% to 28%, it is generally a well-tolerated procedure. Most complications affect local tissue only and typically do not require additional hospitalization. Placement of closed suction drains has been demonstrated to minimize postoperative complications [3–6].

In major head and neck surgery, several studies have demonstrated that the operative time and the blood loss are related to the clinical outcomes and the complications [7, 8].

New technologies and surgical devices focused on reducing operative time, blood loss, operative time, hospital stay, and the rate of complications are emerging and the first results are promising. The harmonic scalpel (HS) is a novel surgical instrument that cuts and coagulates using ultrasonic energy. Influenced by its favourable use in other surgical fields, the HS has, since the 1990s, become more frequently used in ENT surgery, above all in thyroid surgery. Its ability to simultaneously dissect and secure hemostasis ensures a clean, dry surgical field.

The present prospective randomized trial study was designed to evaluate the efficacy and safety of HS use compared with conventional haemostasis (CH) (classic technique of tying and knots, resorbable ligature, and bipolar diathermy) in ND as a part of surgical treatment in oncologic ENT patient. The primary objectives of this study were the reduction of operative time, postoperative pain, intraoperative blood loss and overall drainage volume in neck surgery with the use of the HS. The secondary objective was the comparison between groups of hospital stay and intraoperative and postoperative complications.

## 2. Materials and Methods

Between January 2010 and December 2012, 78 consecutive patients with untreated HNSCC patients underwent ND with primary HNSCC surgery performed by the same team of surgeons. The study was approved by the local Institutional Review Board. All patients were blinded to the surgical technique used and signed an informed consent before enrollment in the trial. The inclusion criteria were (1) age > 18 years, (2) acceptance to participate in the study (signed informed consent form), and (3) scheduled ND with primary HNSCC surgery. The exclusion criteria were (1) preoperative medication including analgesics, corticosteroids, or nonsteroidal anti-inflammatory drugs; (2) coagulation disorders; (3) pregnancy; (4) cases in which the ND specimen could not be separated from the primary tumor; and (5) history of neck irradiation. In all patients, ND was performed first followed by primary tumor resection. ND was separately done by side and also separated from the primary tumor.

After the exclusion of 17 patients, 61 were enrolled and randomly assigned to either the HS group (31 patients in which the operation was performed entirely using the HS and no other haemostatic tool) or the CH group (30 patients in which the operation was performed using CH tools such as the classic technique of tying and knots, resorbable ligature, and bipolar diathermy). Only tumors originating from the oral cavity, oropharynx, hypopharynx, and larynx were included (Table 1).

We performed selective ND (SND) in patients with a clinically node negative neck. According to the primary site of the cancer, SND including level I to III (SND I–III) was performed for the treatment of oral cavity cancer and SND II–IV for oropharynx, larynx, and hypopharynx cancer. The authors performed unilateral or bilateral ND with regard to the status of the primary tumor, such as involvement beyond a midline of primary site.

Patients with a clinically positive neck underwent comprehensive ND (CND). According to Medina's classification and to the American Head and Neck Society (AHNS) and the American Academy of Otolaryngology-Head and Neck Surgery (AAO-HNS) subsequent revisions, we routinely performed modified radical ND (mRND): type I with spinal nerve (SN) preservation, type II with SN and internal jugular vein (IJV) preservation, and type III with SN, IJV, and sternocleidomastoid muscle (SM) preservation (Table 1).

**Figure 1:**
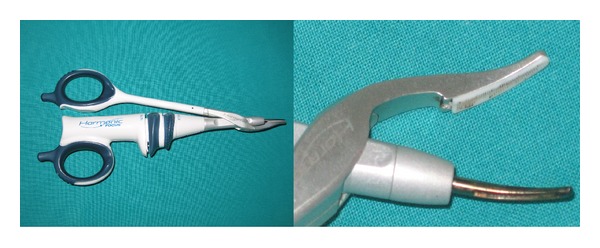
Focus Ultracision harmonic scalpel (Ethicon Endo-Surgery, Inc., Cincinnati, Ohio). The curved blade and the clamp arm with teflon pad of the Focus Ultracision harmonic scalpel used in otolaryngologic surgery.

We used the Focus Ultracision harmonic scalpel (Ethicon Endo-Surgery, Inc, Cincinnati, Ohio) (Figure 1). The HS setup consists of a generator, a hand piece, and a blade. The hand piece contains an ultrasonic transducer that consists of a stack of piezoelectric crystals sandwiched between two metal cylinders under pressure. The transducer is attached to the blade through a mount. The 110-volt generator is a high-frequency switching power supply controlled by a microprocessor that pulses the transducer in the hand piece with AC current. This current allows the transducer to vibrate at its natural harmonic frequency of 55.5 kHz. The blade used most frequently in otolaryngological procedures looks like a curved paddle with a sharp inner beveled side for cutting and a blunt outer radius for coaptive coagulating (Figure 1). The generator can be adjusted from a level of 1 to 5 to increase cutting speed and decrease coagulation by increasing the blade's lateral excursion [9–12].

Outcomes of the study included operative time, intraoperative blood loss, fluid content in the suction balloon (drainage volume) during the first 48 hours after surgery, postoperative pain, hospital stay and incidence of intraoperative (major vessel laceration, major nerve injury, and penetration into adjacent vital structures such as trachea or esophagus), and postoperative complications (hemorrhage, hematoma, seroma, chylous leakage, and neurologic complications).

The operative time and the intraoperative blood loss were recorded starting from the cutaneous incision until the removal of the ND specimen. Suction drainage was used to evaluate the overall amount of blood loss after the procedure and to assess the actual difference between the groups. The drains were removed 48 hours after surgery.

Patients were given acetaminophen, 1000 mg every 8 hours, for the first 24 hours after surgery. Pain assessment was analyzed according to patient responses to a Visual Analogue Scale (VAS) and a verbal response scale (VRS). Anesthesiologists, completely unaware of the surgical instrumentation used during the procedure, collected all data relative to postoperative pain. The VAS consisted of a printed 10 cm horizontal line anchored by the descriptors “no pain” (minimum, on the left end of the scale) and “worst pain imaginable” (maximum, on the right end).

To avoid any setting bias, the clinician always moved the scale's indicator to the horizontal midpoint before the instrument was handed to the patient for a response. The VRS offered 5 options: 0 for no; 1, light; 2, endurable; 3, strong; and 4, unendurable pain. The patients graded their pain at 24 and 48 hours after surgery.

Hospital stay was recorded and compared according to the surgery regarding primary cancer. We classified the primary cancer surgery as wide excision with free-flap reconstruction, wide excision without reconstruction, partial laryngectomy, or total laryngectomy (Table 1).

Intraoperative and postoperative complications were examined and recorded. Postoperative seroma and hematoma were examined for at least 2 weeks postoperatively. We checked the mobility and sensation of tongue at postoperative day 7 after the ND to evaluate the function of the hypoglossal or lingual nerve. The patients whose tongue was resected or whose hypoglossal or lingual nerve was sacrificed during surgery were excluded in this mobility or sensation test.

Diaphragmatic elevation was determined by reviewing the immediate postoperative chest X-ray. The spinal nerve (SN) function was evaluated at 6 months after surgery to see if patients suffered from shoulder syndrome. We assessed the severity of shoulder pain (A 10-point visual analogue scale [VAS]; 1 normal, 10 worst), deformity of shoulder and range of motion score (degree of shoulder abduction; 1: 0°–45°, 2: 45°–90°, 3: 90°–135°, 4: 135°–180°). Patients whose SN was sacrificed during ND were excluded.

Patients were also asked to contact our Department of Otorhinolaryngology after discharge for any postoperative complication such as neck hematoma or seroma and wound infection. All patients gave informed written consent. The results were analyzed using Student's *t*-test and *χ* ^2^ test. A value of *P* < 0.05 was considered statistically significant.

## 3. Results

The demographic characteristics of the patients, the stage of the tumour, and the surgical procedures (primary surgery and type of ND) of both groups are showed in the Table 1. The groups (HS and CH) were homogeneous for age, sex, primary site of the tumor, TNM staging, and type of surgical treatment. In the HS group (*n* = 31), 18 patients underwent bilateral ND; thus, a total of 49 NDs were performed. In the CH group (*n* = 30), 16 patients underwent bilateral ND for a total of 48 NDs. (Table 1).

In the patients undergoing modified RND, the mean operative time was significantly shorter in the HS group (113.9 ± 17.3 minutes) compared with the CH group (149.5 ± 18.7 minutes; *P* < 0.001). The intraoperative blood loss was significantly smaller in the HS group (149.2 ± 26.8 mL versus 201.9 ± 34.2 mL; *P* < 0.001) as well as the total drainage fluid volume (237.4 ± 12.4 mL versus 356.1 ± 24.2 mL, respectively, *P* < 0.001). Similarly, significant results were found in the patients undergoing SND (shorter operative time, smaller intraoperative blood loss, and lower total drainage fluid volume in HS group). According to different surgical treatment of primary site of tumor, no significant difference was found between the two groups concerning the mean hospital stay (Table 2).

Few complications were observed in both groups. One patient in HS group and four patients in CH group had postoperative seroma formation, which were resolved after compressive dressing. Two cases in HS group and three in CH group were treated for a wound infection; all these patients were affected by mellitus diabetes. No postoperative bleeding or hematoma or chylous leakage or vascular and neurologic complications occurred.

According to the VAS and VRS scores, patients of the HS group experienced significantly less postoperative pain compared with patients of the CH group. The differences in VAS scores between the HS and CH groups were statistically significant at 24 and 48 hours (*P* < 0.001). The difference in the VRS score between the groups was statistically significant at 24 and 48 hours after surgery (*P* < 0.001) (Table 2).

Hypoglossal or lingual or phrenic nerve deficit was not observed in all patients. Shoulder syndrome secondary to spinal nerve injury was evaluated at 6 months after surgery. Shoulder pain score and shoulder motion score were not significantly different in both groups (Table 2).

## 4. Discussion

The HS is a new device that has been introduced to surgery during the last decade. It was originally developed for its applications in laparoscopic abdominal surgery but has found its way successfully into the specialty of otolaryngology [13]. The primary applications for the HS in the otolaryngologic literature pertain to its uses for tonsillectomy [9] and thyroidectomy [14]. The use of the HS has also been described in excising cancer of the tongue and soft palate [15], submandibular sialadenectomy [16], parotidectomy [17–19], treating allergic rhinitis by means of inferior turbinate alteration [20], and surgical treatment of rhinophyma [21]. Unlike the variable results described with the use of the HS in tonsillectomy, the literature is consistent concerning the usefulness of the harmonic scalpel in thyroid surgery [22, 23].

ND is a basic procedure in head and neck oncologic surgery. There has been a few reports showing the utility of HS in ND, both RND and SND. In 2008 Salami et al. reported that the use of HS during pharyngolaryngectomy and radical ND (in patients with advanced laryngopharyngeal HNSCC) led to diminished bleeding, shorter operative time, less seroma formation, and better wound healing in the postoperative period [24]. In 2009 Miccoli et al. concluded that the HS during lateral lymphadenectomy (in patients with papillary thyroid cancer with neck metastases) is as safe as conventional technique and may allow shorter operative time, lower lymphatic spillage, and faster decrease of pain [25]. In 2011 Walen et al. performed a prospective randomized controlled trial to determine the impact of the HS on intraoperative blood loss, and operative time in SND (levels I–IV) for HNSCC; they suggest that the HS can reduce blood loss during SND for HNSCC but it has no impact on operative time, postoperative drain output, or complication rate [26]. Recently, in 2012 Shin et al. investigated the safety and the efficacy of HS in patients who underwent ND with primary HNSCC resection, demonstrating a significant reduction of operative time and blood loss in HS group [27].

There is a growing consensus in the literature about the importance of minimizing the intraoperative blood loss and reducing the operative time in patients undergoing major head and neck surgical treatment. New surgical techniques as HS are being developed to enable surgeons to increase their speed and efficiency. The HS is a new device that uses high-frequency mechanical energy to cut and coagulate tissues at the same time. Ultrasonic coagulation achieved by the HS is similar to that of electrocautery in that the ultimate result remains a denatured protein coagulum that coapts and tamponades blood vessels [9]. However, the mechanism by which the proteins become denatured is completely different. Both electrocautery and lasers form the coagulum by heating tissue to denature the protein. The HS denatures protein by using ultrasonic vibration to transfer mechanical energy sufficient to break tertiary hydrogen bonds. At least two mechanisms exist by which the HS cuts: cavitational fragmentation and mechanical cutting. The blade vibrates at 55.5 kHz over a distance of 80 *μ*m [10]. When the temperature reaches 60°C, the proteins begin to denature, transforming from their initial colloidal state into an insoluble gel, which is necessary for vessel coagulation [28]. In a porcine study comparing vessel-sealing systems using various modalities of energy, including the HS, the LigaSure vessel-sealing system (Valleylab, Boulder, Colorado), and two types of bipolar forceps, the HS was found to seal arteries 3.8 mm in diameter on average and veins 9.9 mm in diameter on average. This sealing ability was essentially inferior to that of the other systems. However, the HS showed a smaller area of lateral thermal damage compared to the bipolar cautery [29].

The Ultracision HS has been approved by the United States Food and Drug Administration for the ligation of vessels up to 3 mm in diameter. The last generation of the HS (Harmonic Focus) is even more appropriate since it is approved for closing vessels up to 5 mm in diameter, as the facial artery that is one the largest arteries (2.5 ± 0.1 mm) that have to be ligated during ND [30–34]. Similarly to facial artery, we have performed the correct sealing with HS of other branches of external carotid artery as lingual artery, superior thyroid artery, and occipital artery, without any failure. The control of hemostasis by HS rarely does not occur; in this case we identified the bleeding vessel and we performed a conventional hand-tie ligature.

**Figure 2:**
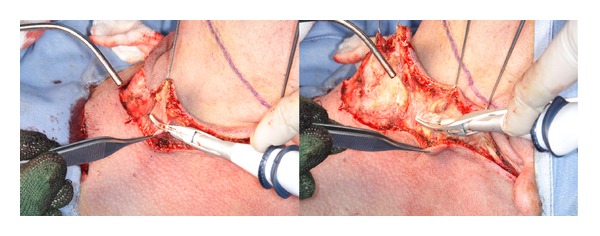
Section of platysma muscle with Focus Ultracision harmonic scalpel during dissection of upper cervical flap.

**Figure 3:**
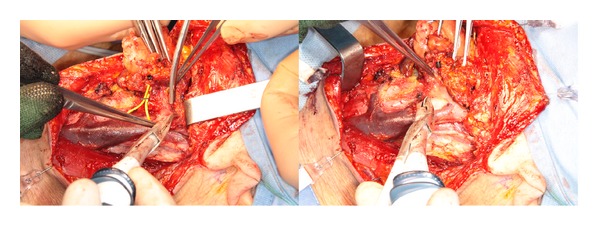
Neck dissection of II-III level with Focus Ultracision harmonic scalpel: plane of the internal jugular vein with sealing of the tributary vessels.

**Figure 4:**
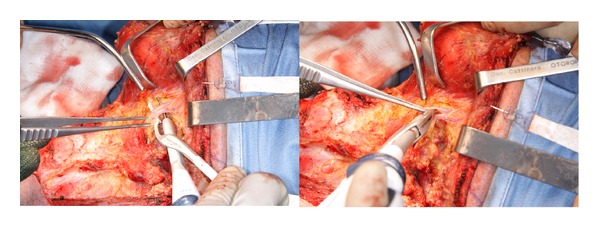
Neck Dissection of II-III level with Focus Ultracision harmonic scalpel: sealing of the facial vessels.

The saved time in HS group was not only related to reducing the conventional hand-tie ligation but also to the ease and speed of dissection, especially in the dissection of upper cervical flap (Figure 2), in the II-III level under the sternocleidomastoid muscle along the plane of the internal jugular vein and in the IV-V level when it is needed to cut the fibrofatty tissue and/or the muscles (sternocleidomastoid and omohyoid) (Figures 3 and 4).

In the literature, there are many papers about the shoulder syndrome and the postoperative pain in ND but our study is the first that demonstrates the significant reduction of algic postoperative symptoms in ND using HS. During ND, the surgeons should pay attention to the integrity and the saving of many neural structures (spinal nerve, hypoglossal nerve, lingual nerve, and phrenic nerve) that, if damaged, would complicate the postoperative course of the patient. Even dissection with preservation of the nerves may lead to sequelae. So, postoperative pain of neck and shoulder syndrome is usual in 30% to 70% of patients after ND, depending on the procedure extent and on how symptom severity is defined [35–37]. Our study shows that it is relevant the use of HS in reducing the postoperative algic symptoms but not the shoulder syndrome. Experimental studies have proved that the thermal spread of HS is limited to 0–2 mm beyond the tissue grasped within the forceps of the device [34, 38]. A possible explanation of our results is that the HS causes reduced tissue injury, with no neuromuscular stimulation, as would be induced by electrocautery or other devices.

Although HS is considered a reliable and safe tool, potential disadvantages have been reported in thyroid surgery as the thermal injury to the surrounding tissues, the surgical instrument fractures due to the heat generated by the device, and the expense of the disposable handpieces [39–41]. Recently, Lombardi et al. have demonstrated a cost-effectiveness benefit for the use of HS [42]. However, these studies do not concern the use of HS in neck dissection.

Even if our study design was correct, there are some limitations to this work. This is a study performed by the same team of surgeons in three ENT departments, but a multicenter research could limit any surgical technique bias caused by evaluating surgeons from few centers. Moreover, it is possible that we may have underestimated the intraoperative blood loss because the effect of the hemodilution performed during the surgery was not considered. Furthermore, another limitation of this study is that patients with different types and extents of surgery were all included in the work; therefore, we have stratified the patients according to surgical procedure to minimize the potential bias. At last, we did not carry out an economic evaluation of the use of HS in neck dissection. In our opinion, these limitations do not invalidate our results and our conclusions about the efficacy and safety of HS-aided neck dissection.

## 5. Conclusions

This study demonstrated the effectiveness of using HS in neck dissection compared to conventional surgical haemostasis instruments. The HS is a reliable and safe tool for reducing intraoperative blood loss, postoperative pain, and operative time in patients undergoing ND for HNSCC and above all in the cases where minimizing blood loss and reducing surgical time are notable for the clinical outcome. Multicenter randomized studies need to be done to confirm the safety and the advantages of this technique and to evaluate the cost-benefit ratio in neck dissection procedures.

## Figures and Tables

**Table 1:** Demographic characteristics, stage of the tumour, and type of surgical procedure in harmonic scalpel (HS) and conventional hemostasis (CH) groups.

	HS group (*n* = 31)	CH group (*n* = 30)	Total (*n* = 61)
Age (years) (range)	61.2 (35–79)	60.4 (38–81)	
Sex (M/F)	26/4	27/4	53/8
Primary site			
Oral cavity	4	5	9 (13%)
Oropharynx	10	8	18 (29%)
Larynx	9	11	20 (32%)
Hypopharynx	8	6	14 (26%)
T stage			
T1	4	5	9 (13%)
T2	18	17	35 (58%)
T3	7	6	13 (22%)
T4	2	2	4 (7%)
N stage			
N0	13	14	27 (44%)
N1	8	7	15 (25%)
N2	6	5	11 (18%)
N3	4	4	8 (13%)
Primary surgery			
Wide excision	13	9	22 (36%)
Free-flap reconstruction	9	10	19 (31%)
Partial laryngectomy	8	9	17 (28%)
Total laryngectomy	1	2	3 (5%)
Neck dissection			
mRND	24	22	46 (47%)
Type I	6	7	13 (14%)
Type II	7	6	13 (14%)
Type III	11	9	20 (21%)
SND (I–III)	12	14	26 (27%)
SND (II–IV)	13	12	25 (26%)

**Table 2:** Operative and postoperative data in harmonic scalpel (HS) and conventional hemostasis (CH) groups.

	HS group (*n* = 31)	CH group (*n* = 30)	*P* value
Operative time (mean ± SD), min			
mRND	113.9 ± 17.3	149.5 ± 18.7	<0.001
SND	57.5 ± 14.9	86.1 ± 20.6	<0.001
Intraoperative blood loss, mL			
mRND	149.2 ± 26.8	201.9 ± 34.2	<0.001
SND	61.7 ± 20.1	97.9 ± 31.5	<0.001
Total drainage fluid volume, mL			
mRND	237.4 ± 12.4	356.1 ± 24.7	<0.001
SND	129.3 ± 28.1	238.9 ± 30.6	<0.001
Postoperative pain			
VAS at 24 h			
mRND	4.89 ± 1.07	6.82 ± 1.43	<0.001
SND	4.27 ± 1.51	6.35 ± 2.04	<0.001
VAS at 48 h			
mRND	1.98 ± 0.96	3.81 ± 1.37	<0.001
SND	1.76 ± 0.82	3.99 ± 1.45	<0.001
VRS at 24 h			
mRND	2.91 ± 1.05	4.15 ± 1.94	<0.001
SND	2.84 ± 1.19	4.27 ± 1.59	<0.001
VRS at 48 h			
mRND	1.06 ± 0.87	2.73 ± 0.71	<0.001
SND	0.95 ± 0.44	2.85 ± 1.03	<0.001
Mean hospital stay, days			
Wide excision	9.2 ± 2.5	10.1 ± 2.8	0.467 NS
Free-flap reconstruction	24.9 ± 3.3	27.1 ± 3.4	0.579 NS
Partial laryngectomy	25.1 ± 2.9	28.0 ± 2.7	0.418 NS
Total laryngectomy	16.7 ± 2.1	17.4 ± 3.0	0.799 NS
Shoulder syndrome			
Pain score (VAS)			
mRND	3.46 ± 0.22	3.35 ± 0.15	0.924 NS
SND	1.15 ± 0.18	1.27 ± 0.33	0.871 NS
Motion score (VAS)			
mRND	2.14 ± 0.51	2.21 ± 0.47	0.916 NS
SND	1.18 ± 0.44	1.06 ± 0.12	0.949 NS
